# Cell-free DNA mutations as biomarkers in breast cancer patients receiving tamoxifen

**DOI:** 10.18632/oncotarget.9727

**Published:** 2016-05-30

**Authors:** Maurice P.H.M. Jansen, John W.M. Martens, Jean C.A. Helmijr, Corine M. Beaufort, Ronald van Marion, Niels M.G. Krol, Kim Monkhorst, Anita M.A.C. Trapman-Jansen, Marion E. Meijer-van Gelder, Marjolein J.A. Weerts, Diana E. Ramirez-Ardila, Hendrikus Jan Dubbink, John A. Foekens, Stefan Sleijfer, Els M.J.J. Berns

**Affiliations:** ^1^ Department of Medical Oncology and Cancer Genomics, Erasmus MC Cancer Institute, Rotterdam, The Netherlands; ^2^ Department of Pathology, Erasmus MC Cancer Institute, Rotterdam, The Netherlands; ^3^ Cancer Computational Biology Center, Rotterdam, The Netherlands

**Keywords:** breast cancer, tamoxifen therapy, targeted next generation sequencing, cell-free DNA, disease progression

## Abstract

The aim was to identify mutations in serum cell-free DNA (cfDNA) associated with disease progression on tamoxifen treatment in metastatic breast cancer (MBC). Sera available at start of therapy, during therapy and at disease progression were selected from 10 estrogen receptor (ER)-positive breast cancer patients. DNA from primary tumor and normal tissue and cfDNA from minute amounts of sera were analyzed by targeted next generation sequencing (NGS) of 45 genes (1,242 exons). At disease progression, stop-gain single nucleotide variants (SNVs) for *CREBBP* (1 patient) and *SMAD4* (1 patient) and non-synonymous SNVs for *AKAP9* (1 patient), *PIK3CA* (2 patients) and *TP53* (2 patients) were found. Mutations in *CREBBP* and *SMAD4* have only been occasionally reported in breast cancer. All mutations, except for *AKAP9*, were also present in the primary tumor but not detected in all blood specimens preceding progression. More sensitive detection by deeper re-sequencing and digital PCR confirmed the occurrence of circulating tumor DNA (ctDNA) and these biomarkers in blood specimens.

## INTRODUCTION

It is increasingly appreciated that the genetic make-up of tumors forms one of the main determinants for outcome to systemic treatments in cancer patients [1]. There is also accumulating evidence that primary tumor characteristics can greatly differ from those of the metastases [2]. This may underlie the relatively poor association of molecular characteristics of primary tumors with outcome in MBC. It is therefore likely that genetic variants important for treatment decision making should be determined in specimens from metastatic tumor rather than from primary tumor tissue. In addition, the genetic constitution of a tumor lesion is not fixed but constantly changes, in particular under treatment pressure. As novel DNA mutations can cause resistance to systemic treatments, longitudinal monitoring of these mutations during treatment is crucial to detect resistance at an early stage and, if possible, to adjust treatment based on the emerging mutations.

DNA from primary and metastatic tumor cells can be detected as cfDNA in the peripheral blood of cancer patients. This cfDNA is therefore a very attractive tool to establish mutational changes occurring in tumor cells in a minimal invasive manner. Its great promise in this respect was recently reviewed [3]. For example, in patients with metastatic *KRAS* wild-type colorectal cancer treated with an anti-EGFR antibody, blood analyses showed that the appearance of *KRAS* mutants, conferring resistance against anti-EGFR antibodies, preceded progressive disease with up to 10 months [4]. Likewise, in breast cancer patients mutations in the estrogen receptor (ESR1) have been hardly detected in primary tumors but are currently frequently reported in plasma from patients with metastatic disease that acquired resistance to aromatase inhibitor therapy [5–8]. In the current study, we aimed to identify tumor-specific mutations in cfDNA that associate with disease progression on tamoxifen in MBC.

## RESULTS

### Detection of DNA changes

**Figure 1:**
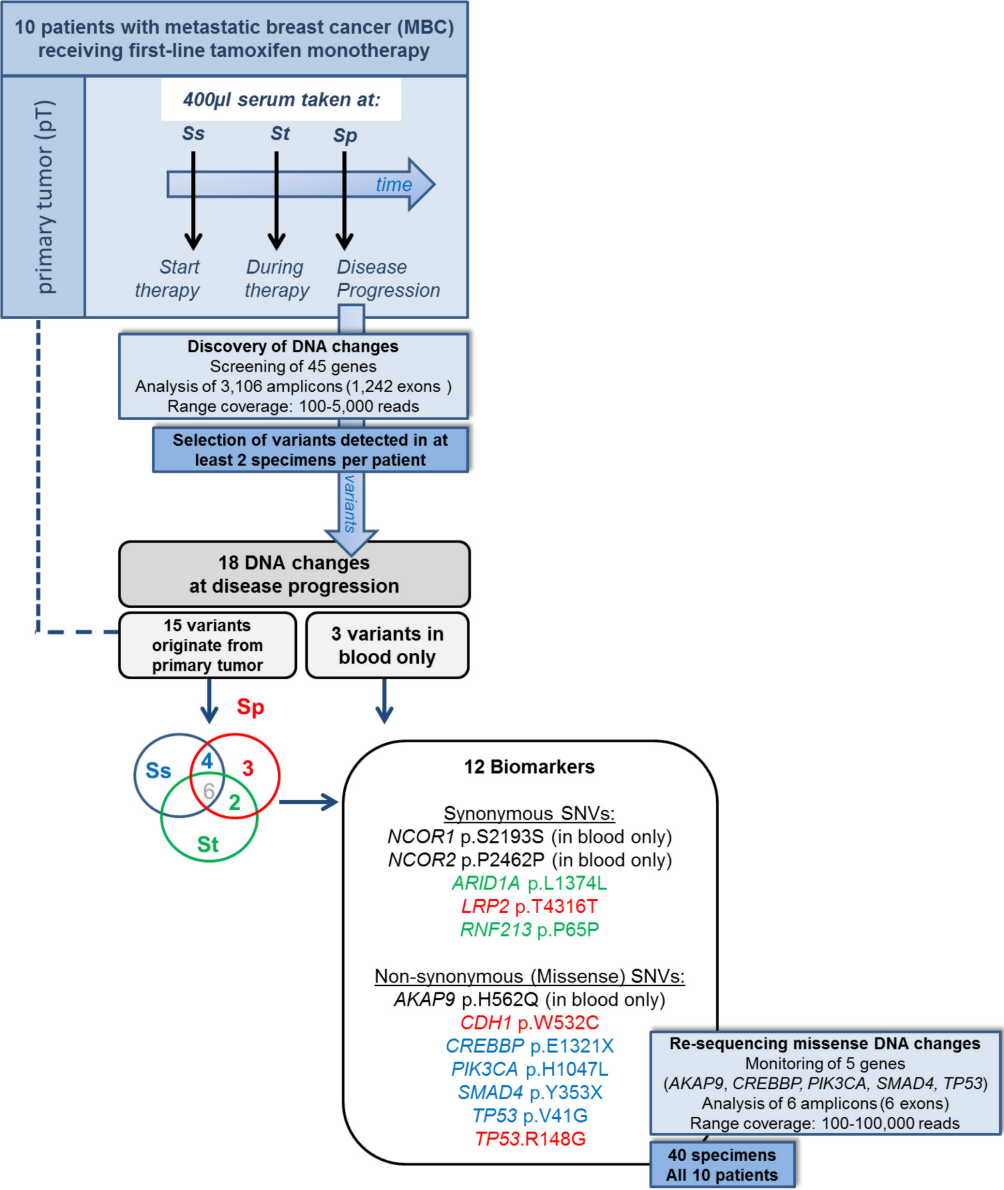
Study design and discovered DNA changes Targeted ion-PGM (re-)sequencing was performed on DNA isolated from primary tumors and blood specimens from 10 metastatic breast cancer patients who received tamoxifen as first-line therapy. Cell-free DNA (cfDNA) was isolated from 400 μl serum taken at start (Ss), during therapy (St) and at disease progression (Sp). Analysis revealed 12 biomarkers including 9 single nucleotide variants (SNVs) detected at progression and in primary tumor but not in all preceding blood specimens. The SNVs originating from the primary tumor are presented in red when only seen at Sp, in green when seen at Ss and Sp, and in blue when seen at St and Sp.

As we were interested in cfDNA mutations that potentially associate with treatment outcome in a particular patient, we characterized DNA changes in serum taken at disease progression in 10 MBC patients who received first-line tamoxifen (Supplementary Table S1). After applying our selection criteria on called variants, 18 cfDNA changes were identified at disease progression which were not detected in normal tissue DNA nor reported by the 1000 Genome database. Of these, 3 variants were only seen in blood specimens and not in the corresponding primary tumor whereas 15 variants were also detectable in the primary tumor. Twelve DNA changes in 6 patients were shown to associate with treatment outcome (Figure 1). Of these, 9 tumor-specific DNA changes were present at disease progression and in the primary tumor but not in all blood specimens preceding progression (Table 1).

**Table 1:** 12 cfDNA mutations identified as biomarkers in 6 metastatic breast cancer patients receiving first-line tamoxifen therapy^1^

Results obtained in cfDNA^2^ and calculated pel 1 ml serum													
		Primary Tumor (pT)			At start therapy (Ss)			During therapy (St)			At disease progression (Sp)		
Gene	Amini Acid change	DNA input (in ng)	Variant frequency (in %)	number mutant copies per 1 ng serum	yield cfDNA (in ng)	variant frequency (in %)	number mutant copies per 1 ml serum	yield cfDNA (in ng)	Variant frequency (in %)	number mutant copies per 1 ml serum	yield cfDNA (in ng)	Variant frequency (in %)	number mutant copies per 1 ml serum
**Patient 1^3^**		16.6			30.8			17.0			16.3		
*NCOR2*	P.P2426P		0	0		0	0		2	103		3	148
*AKAP9*	P.H562Q		0	0		16	1493		17	876		2	99
**Patient 6**		15.8			23			29.7			24		
*RNF213*	P.P65P		0	0		0	0		0	0		2	145
*CDH1*	P.W532C		0	0		0	0		0	0		1	73
*CREBBP*	p.E1321X		23	65		2	139		0	0		4	291
**Patient 7**		14.8			14.8			7.3			17.5		
*PIK3CA*	p.H1047L		25	76		2	89		0	0		7	371
*SMAD4*	P.Y353X		17	52		8	358		0	0		4	212
*TP53*	p.V41G		35	106		3	134		0	0		2	106
**Patient 8**		13.0			17.5			24.3			113.8		
*NCOR1*	P.S2193S		0	0		0	0		4	294		4	1379
*LRP2*	P.T4316T		10	35		0	0		0	0		2	689
*RNF213*	P.P65P		2	7		0	0		3	220		3	1034
*CDH1*	pW532C		2	7		0	0		0	0		1	345
*TP53*	P-R148G		35	121		0	0		0	0		3	1034
**Patient 9**		9.8			34.0			21.0			10.8		
*ARID1A*	P.L1374L		57	261		50^4^	5152		60	3818		48	1564
**Patient 10**		12.2			6.8			10.5			10.3		
*CDH1*	P.W532C		3	11		0	0		0	0		3	93
*PIK3CA*	P.H1047L		43	158		0	0		0	0		0	0
*TP53*	P.R148G		0	0		0	0		2	64		0	0

1 No DNA changes were identified at disease progression and detected in corresponding primary tumor and/or blood specimens at earlier time-points for patients 2 ro 5.

2 Input was 400 μl serum.

**Figure 2:**
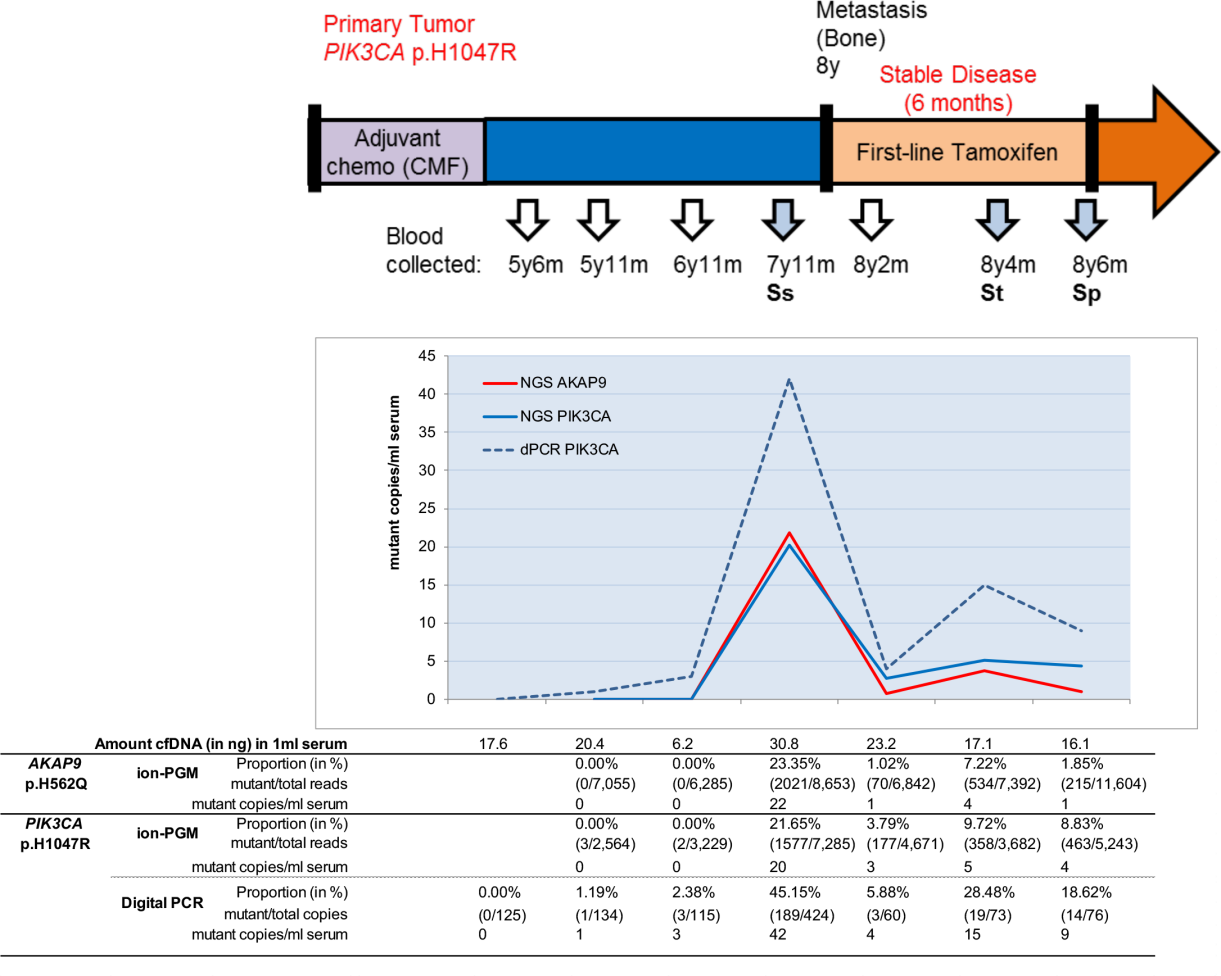
cfDNA missense mutations and disease development The *AKAP9* and *PIK3CA* mutations of patient 1 were evaluated in blood specimens during the course of disease. Sera collected five years after clinical diagnosis of breast cancer were evaluated by ion-PGM resequencing and for *PIK3CA* in duplicate by digital PCR (dPCR). The p.H1047R mutation was observed at low magnitude in blood taken five years after diagnosis of primary disease and already two years before radiological diagnosis of metastatic lesions. Although sometimes low numbers of *PIK3CA* mutant copies were detected, all were independently observed after ion-PGM resequencing and in two separate digital PCR reactions. All proportions except for the sample at 5y11m were above the limit of detection.

3 Patient 1 had a PIK3CA p.H1047R mutation not called by standard ionPGM settings, but which was confirmed by re-sequencing and digital PCR (See also figure 2 and additional Table Al).

4 The ARIDIA SNV was observed at a mutation frequency of 50% in specimen Ss but not called due to strand bias, i.e. this SNV was detected in 7 forward and 289 reverse strand reads.

### Pathogenic somatic single nucleotide substitutions

The 12 DNA changes included 5 synonymous and 7 non-synonymous SNVs. Almost all algorithms predicted the missense SNVs for *CDH1*, *PIK3CA* and *TP53* (p.V41G; p.R148G) as pathogenic and for *AKAP9*, *CREBBP* and *SMAD4* predominantly as not pathogenic (Supplementary Table S2). Moreover, the SNVs in *PIK3CA* and *TP53* have been reported as cancer-specific mutations.

### Re-sequencing at 1% detection limit of missense SNVs

Next the identified non-synonymous SNVs were selected for re-sequencing since only these translate into amino acid changes, which might alter the biological function of the encoded protein and as a result might affect clinical outcome. All specimens were re-sequenced for 1 missense SNV found in blood only and 5 missense SNVs found in primary tumors at diagnostic levels, i.e. higher than 10%. The re-sequencing confirmed the initial results for *AKAP9* p.H562Q in patient 1, *CREBBP* p.E1321X in patient 6, *SMAD4* p.Y353X, *TP53* p.V41G, and *PIK3CA* H1047L in patient 7, and *TP53* p.R148G in patient 8. Moreover, *PIK3CA* exon 20 re-sequencing identified another SNV, i.e. p.H1047R. This SNV was seen at disease progression and all other blood specimens of patient 1 and in the primary tumors of patients 1, 2, and 7 (Supplementary Table S3).

### Digital PCR evaluation at 0.05% detection limit of *PIK3CA* mutations

The re-sequencing results for *PIK3CA* were verified by digital PCR using mutation-specific assays (Supplementary Table S3). The p.H1047L mutation was evaluated and confirmed in all specimens of patients 7 and 10, but was additionally seen in serum at start of therapy of patient 10. The p.H1047R mutation was evaluated in all primary tumors as well as in blood specimens of patients 1 and 2. Digital PCR confirmed the occurrence of this mutation in all evaluated blood specimens and primary tumors, except in the primary tumor of patient 7.

### cfDNA mutations and disease development

For patient 1 additional blood specimens available between diagnosis of primary tumor and metastatic lesions were evaluated by NGS and digital PCR (Figure 2). Re-sequencing demonstrated *AKAP9* and *PIK3CA* mutations at similar magnitudes in blood specimens taken around the occurrence of metastatic lesions. It also detected PIK3CA mutant reads in blood preceding the metastasis, however, in less than 10 reads. Digital PCR confirmed this *PIK3CA* mutation in blood taken 6 years after diagnosis of primary disease but two years before diagnosis of metastatic lesions. At the time metastatic lesions were recognized, the blood had a large number of copies with this mutation, which dropped after 2 months of first-line tamoxifen therapy, but increased towards therapy resistance after 6 months treatment.

## DISCUSSION

This exploratory study is to our knowledge the first to report on sequential monitoring of serum cfDNA in a homogenous setting of MBC-patients receiving first-line tamoxifen therapy. In total 12 variants for 6 patients were identified in cfDNA at disease progression, including 3 variants that were only found in blood specimens but not in the primary tumor and 9 variants detected in corresponding primary tumor but not in all blood specimens preceding progression. Because of their putative biological relevance, we confirmed the identified missense mutations by re-sequencing and by digital PCR.

Out of these, missense mutations in *PIK3CA*, *TP53, SMAD4* and *CREBBP* were present both at time of progression and in the primary tumor. COSMIC reported mutations in breast cancer most frequently in *PIK3CA* and *TP53* while occasionally in *CREBBP* and *SMAD4* with our variants for the latter two genes not earlier described. Mutations in all these genes have been found in hormone-resistant breast cancer [9]. The presence of *PIK3CA* and *TP53* mutations in longitudinally collected blood specimens correlated with treatment outcome to PI3K-inhibitors and aromatase inhibitors and was associated with the clinical course of disease [10, 11]. *CREBBP* and *SMAD4* encode proteins that bind to ER as co-activator [12] and co-repressor [13], respectively, suggesting a putative role in endocrine therapy resistance [13, 14]. However, the effect of the *CREBBP* stop-gain mutation revealed in our study remains to be established. The *SMAD4* p.Y353X stop-gain mutation resides within the MH2 domain, a mutational hotspot [15] related to a loss of function, detrimental for TGFb signaling, and poor disease outcome [16]. Importantly, above variants at time of progression likely reflect only tumor load in the blood. The *AKAP9* p.H562Q missense mutation was not seen in the primary tumor and might have been missed due to tumor cell heterogeneity. Alternatively, this mutation might originate from metastatic lesions or acquired due to treatment pressure. Variants in *AKAP9* have been described repeatedly in COSMIC and as SNPs associated with increased breast cancer risk [17], but the particular variant found here has not yet been reported. It is currently unknown whether, and if so, to which extent, all missense mutations actually contribute to resistance against tamoxifen. Many of the mutations are probably bystander mutations due to genomic instability. Therapy resistance, however, may select for tumor cells with specific mutations adapting these cells to the hostile environment, resulting in the survival of fittest and ultimately driving tumor progression [18].

Our study differs from previous studies using ion-PGM targeted NGS on cancer tissue and liquid biopsies to identify tumor-specific DNA changes. Earlier studies [19–21] evaluated 200 amplicons of the commercially available hotspot cancer panel whereas we examined fifteen-fold more amplicons. These studies detected mutations in both plasma and tissue for 27 of 34 cancer patients [20], or like our study, in half of the cancer patients [19, 21] and evaluated plasma specimens collected within a 16 month time-frame after primary tumor tissue was obtained. We instead sequenced and identified mutations in minute cfDNA amounts isolated from serum collected at least 3 years after diagnosis of primary disease. Mutations in low DNA amounts might be missed due to the limited number of genomic equivalents present and corresponding higher limit of detection. Retrospective studies, such as our own, collected mainly limited blood quantities which will often result in minute cfDNA amounts available for analysis. Furthermore, our serum samples have been stored at −80°C for more than 18 years, demonstrating that long-term stored routinely collected sera are suitable for cfDNA isolation and subsequent molecular characterization.

To define ctDNA mutations in blood associated with disease progression on tamoxifen treatment, we screened for biomarkers seen at progression and the corresponding primary tumor but not in all preceding blood specimens in a particular patient. The mutation detection in blood depends on cfDNA quantities, with these quantities changing in time and reflecting tumor load in the course of disease. Our study showed overall no significant differences in blood cfDNA yields at different time-points, however, most mutations were detected in patients with the highest DNA yields at progression. Deeper re-sequencing confirmed the presence of the 6 missense SNVs in specimens of individual patients in which they were initially reported and absence from those which initially lack them. It also discovered an additional *PIK3CA* mutation, and examination of sequence reads revealed that the p.H1047R mutation was originally present in respective specimens but not called due to stringent settings, indicating that current thresholds are suboptimal for rare variant detection in cfDNA [19, 22]. Digital PCR independently identified both *PIK3CA* mutations in blood specimens at higher frequencies than revealed by NGS and in additional specimens and as proof-of-principle even in blood taken years before diagnosis of the metastatic lesions.

In conclusion, our study demonstrates that targeted ion-PGM sequencing of cfDNA is applicable to discover mutations in archived serum samples. Deeper re-sequencing and digital PCR analyses enables more sensitive detection and monitoring of specific mutations in sequential blood specimens even in samples stored for over 18 years and in minute amounts of cfDNA. Further studies are warranted to investigate whether detection of ctDNA in tamoxifen-treated metastatic breast cancer patients can be used to detect disease progression at an early stage and whether the identified variants play a role in tamoxifen resistance.

## MATERIALS AND METHODS

Materials and methods are described briefly below, details are found in the appendix.

### Patient and sample collection

This retrospective study investigated fresh frozen primary tumor tissue and sequential sera taken from 10 MBC patients who received tamoxifen as first-line therapy for distant metastatic disease (Figure 1). Blood specimens were selected at start of tamoxifen therapy (Ss), during therapy (St), and at disease progression (Sp). From 6 patients formalin fixed paraffin embedded (FFPE) macro-dissected normal tissue was available and analyzed. The study was approved by the medical ethics committee (MEC 02.953), performed according to the Code of Conduct of Medical Scientific Societies (www.federa.org/codes-conduct) and followed REMARK guidelines where possible [23]. Clinicopathological characteristics are presented in Supplementary Table S4.

### DNA isolation, quantification, and sequencing

DNA from tumor and normal tissue specimens was extracted as described previously [24, 25]. The MagnaPure Compact nucleic acid isolation kit (Roche Diagnostics) was applied to isolate cfDNA from 400 μl serum. DNA yields and concentrations were quantified with a Qubit^®^ 2.0 fluorometer (Thermo Scientific). The cfDNA input amounts of each sample were used to establish the genomic equivalents and limits of detection for subsequent molecular analyses (Supplementary Tables S5 and S6). Semiconductor sequencing was performed using the Ion Torrent Personal Genome Machine (Ion-PGM) and consumables, kits, software packages and protocols provided by the manufacturer (Thermo Scientific). Briefly, 10 ng tissue DNA and minute amounts cfDNA (range: 165-573 pg) were used as input for library preparation and sequenced with a custom-made gene panel. Ion AmpliSeq Library Preparation Kit 2 and Ion PGM Template OT2 200 kit were applied to generate libraries and templates, respectively. Ion Sequencing Kit v2 was used for sequencing on an Ion 318 chip.

### Custom gene panel

The 45-gene panel (Supplementary Table S2) included the most frequently mutated genes for breast, colon, prostate and ovarian cancer reported in the catalogue of somatic mutations in cancer (Cosmic Release 67; http://cancer.sanger.ac.uk/cancergenome/projects/cosmic/). Thirty-nine genes were sequenced for all exons, 6 oncogenes for hotspot exons only. In total 3,106 amplicons (i.e. 1,242 exons; ∼255kb) were sequenced up to a read depth of 5,000x.

### Bio-informatics for variant detection and evaluation

Raw data analyses, base calling and alignment were performed using Torrent Suite v4.0. Somatic low stringency filtering was applied in Variant Caller v4.16 (VC) to detect DNA changes when compared to reference genome hg19 (build 37). Variants were annotated by a custom pipeline including ANNOVAR (openbioinformatics.org/annovar) within Galaxy (galaxyproject.org). Only exonic variants with frequencies of 1% or higher and above the cfDNA-specific limit of detection were selected. Uniquely identified variants and those found in sequenced normal DNA or reported within the 1000-Genome database were excluded. These variants had to be sequenced without strand bias at a read depth of 100x or more and showing at least 10 mutant reads. Integrative Genomics Viewer (IGV) (http://www.broadinstitute.org/igv) was used for manual examination. Identified SNVs were evaluated with different *in silico* algorithms to predict the pathogenicity of the SNV on protein function. These tools are embedded in ANNOVAR and included SIFT, PolyPhen2, MutationTaster, FATHMM, GERP++, SiPhy and PhyloP [26].

### Re-sequencing and digital PCR analysis

Exons of selected non-synonymous SNVs were re-sequenced for all specimens by ion-PGM after independent library preparation up to 100,000 reads depth, and evaluation was performed similar to the initial analysis. The *PIK3CA* genotype was verified with Taqman p.H1047L- and p.H1047R-specific assays and the QuantStudio™ 3D Digital PCR system (Thermo Scientific). Reaction mixtures, including tumor or serum DNA and QuantStudio™ 3D Digital PCR Master Mix, were loaded on digital PCR chips with 20,000 wells, and cycled under standard conditions for 40 cycles. QuantStudio™ 3D analysisSuite™ determined the proportion mutant and wild-type templates.

## SUPPLEMENTARY MATERIALS


















