# Supplementary material for: Cell-free DNA mutations as biomarkers in breast cancer patients receiving tamoxifen
Source: Oncotarget. 2016 May 30;7(28):43412–8. doi: 10.18632/oncotarget.9727 (PMC5190033; doi:10.18632/oncotarget.9727)
Supplement: Supplementary file 1 [file oncotarget-07-43412-s001.pdf]

Cell-free DNA mutations as biomarkers in breast cancer patients receiving tamoxifen

Supplementary Materials

MATERIALS AND METHODS

Patient and sample collection

This retrospective study investigated fresh frozen primary tumor tissue and sequential sera taken from 10 metastatic breast cancer patients who received tamoxifen as first-line therapy for metastatic disease. For 6 patients formalin fixed paraffin embedded (FFPE) normal tissue was also analyzed. The patients were selected for the presence of serum taken within two weeks at start tamoxifen therapy (Ss), at therapy response (St), and at disease progression (Sp). Blood specimens were stored in our sera bank at −30°C ,and for the 10 patients of this study collected between June 1992 until August 1997 (Table 1).

Clinicopathological characteristics of the patients are described in detail in Supplementary Table A4. Follow-up for these patients ranged from 3 to 12 years. All patients receiving tamoxifen had stable disease for 6 up to 36 months, except patient 5 who had partial response for 13 months. None of the patients had a distant metastasis at time of initial diagnosis. The median time between initial diagnosis and occurrence of distant metastasis was 78 months (range: 29-124 months). At start of therapy for metastatic disease the median age was 57 years (range: 52–72 years), 8 patients were postmenopausal. Six patients, including one premenopausal patient, received adjuvant chemotherapy either anthracycline-based ($N = 2$) or non-anthracycline-based ($N = 4$) therapy. All patients had ER-positive (> 10 fmol/mg cytosolic protein; median 50; range 17–412) primary tumors, eight tumors were progesterone receptor positive (> 10 fmol/mg cytosolic protein; median 76; range 23-635) as well. Southern blot analyses of *HER2* available for 5 tumors revealed in 2 tumors an *HER2* amplification [1].

DNA isolation and quantification

The DNA from fresh frozen tumor tissue specimens and from macro-dissected FFPE normal tissue was extracted as described previously [1, 2]. The MagnaPure Compact nucleic acid isolation kit I (Roche Diagnostics) was used to isolate cfDNA from 400 µl serum according to the manufacturer's manual and to standard operating procedures. A Qubit® 2.0 fluorometer (Thermo Scientific) was used to quantify DNA yields and concentrations.). The cfDNA input amounts of each sample were used to establish the genomic equivalents and limits of detection for subsequent molecular analyses (Supplementary Tables A5 and A6). The cfDNA levels of 400 µl serum were on average 1.5 times higher in sera taken at disease progression (Sp = 11.03 ng; range: 4.16–45.50 ng) than during treatment (St = 7.07 ng; range: 2.96–11.92 ng) or at start of therapy (Ss = 8.48 ng; range: 2.70–19.20 ng). Per patient, increased cfDNA levels at disease progression compared to start of therapy were only observed for 4 patients.

Next generation sequencing

An Ion Torrent Personal Genome Machine was used for semiconductor sequencing of normal, tumor and serum-derived DNA applying consumables, kits, software packages and protocols of the manufacturer (Thermo Scientific). DNA was sequenced for a custom Gene panel (see below). Briefly, Ion AmpliSeq Library Preparation Kit 2 and Ion PGM Template OT2 200 kit were applied to generate libraries and templates, respectively, and Ion Sequencing Kit v2 was used for sequencing on an Ion 318 chip. In total 3106 amplicons comprising 1,242 exons (~255kb) were sequenced at 200 to 5,000 reads depth coverage. Thirty-nine genes were sequenced for all exons, whereas 6 oncogenes (*PIK3CA, EGFR, AKT, BRAF, KRAS, CTNNB1*) were analyzed for their hotspot exons only. Standard amounts of 10 ng tumor or normal tissue DNA and minute amounts of cfDNA (median 316 pg; range: 165-573 pg) were used as input for the library preparation. The library preparation for the minute cfDNA input was adjusted from the standard 18 PCR cycli to 21 PCR cycli.

Custom gene panel

The custom panel included 45 most frequently mutated genes for breast, colon, prostate and ovarian cancer reported in the catalogue of somatic mutations in cancer (Cosmic Release 67; http://cancer.sanger.ac.uk/cancergenome/projects/cosmic/). Detailed information for the 45 genes is presented in Supplementary Table A7. According to Ingenuity Pathway Analysis (IPA) for breast cancer, the genes of this panel connected in particular to two multigene networks consisting of 17 and 10 genes, respectively (Supplementary Figure A1). The first network was related to cell cycle and included

AKT1, ATM, BRCA1, CDH1, CHEK2, CREBBP, CTNNB1, EGFR, ERBB2, MLH1, NCOA3, NCOR1, NCOR2, PIK3CA, PIK3R1, RB1, and *TP53*. The second network was coupled to gene expression and contained *FBXW7, GATA3, KMT2A, KMT2D, MED12, NCOR2, PPP2R1A, PTEN, RUNX1,* and *SMAD4.* A subset of the genes was (also) linked in KEGG to signal transduction pathways such as WNT-signaling (*APC, CTNNB1, FBXW7),* RAS/MAPK-signaling (*BRAF*, *KRAS, MAP2K4, MAP3K1),* P53-signaling *(ATM, CHEK2, PTEN, TP53),* and PI3K/AKT-signaling *(AKT1, PIK3CA, PIK3R1, PTEN).*

Bio-informatics for DNA variant detection and evaluation

The Torrent Suite v4.0 (Thermo Scientific) was used for raw data analyses, base calling and alignment. Variant Caller v4.16 (VC, Thermo Scientific) was applied to detect DNA sequence alterations. Annotation of the variants was performed by a custom pipeline including ANNOVAR (openbioinformatics.org/annovar) in a Galaxy (galaxyproject.org) environment [3–7]. For the initial VC analysis of each normal, tumor and serum DNA-sample, PGM somatic low stringency filter settings were applied to detect DNA changes when compared to the reference genome (hg19; build 37). Subsequently, the identified DNA alterations were collected for each tumor and serum sample and combined for all patients, then re-analyzed with the VC to obtain information per DNA change for all patients. All variants were annotated to different public available databases such as the TCGA-breast cancer database, and in ANNOVAR embedded databases including Cosmic, 1000 genomes, and SNP databases. Informative DNA changes were visually examined using Integrative Genomics Viewer (IGV) software (http//www.broadinstitute.org/igv) [8].

This study selected exonic DNA changes with variant frequencies at 1% or higher, i.e. detection threshold of ion-PGM. Unique variants, i.e. detected in only one single specimen, and variants detected in (matched) normal DNA or reported in the 1000 Genome database were excluded for further analyses. In addition, only variants were selected when sequenced in two specimens for a particular patient and with less than 10% bias between forward and reverse strand reads, at 100 reads depth or more, and with at least 10 reads containing the DNA change.

Re-sequencing of single nucleotide substitutions

The exons in which informative non-synonymous and stop-gain single nucleotide variants (SNVs) were discovered were re-sequenced at a deeper read level (average 13,000 reads depth coverage) by ion-PGM. After independent library preparation of each specimen, all tumor DNAs and serum cfDNAs were sequenced. For the re-sequencing data, VC analysis was performed with similar detection criteria described above for the initial analysis.

Digital PCR evaluation of single nucleotide substitutions

For independent verification of identified exon 20 mutations in *PIK3CA*, Taqman mutation specific assays (Thermo Scientific: PIK3CA_775, AHAPAVCD; PIK3CA_776, AHLJOTP) were applied on the QuantStudio™ 3D Digital PCR system (Thermo Scientific), according to the manufacturer specifications. Briefly, the PCR reaction mix is partitioned over a large number of wells to allow accurate absolute quantification of the number of nucleic acid templates. The number of template copies present in the sample is calculated using the number of wells with amplification product and applying the Poisson distribution assumption. Reaction mixtures included QuantStudio™ 3D Digital PCR Master Mix together with 10 and 30 ng DNA of the primary tumor and up to 2 ng serum cfDNA. Serum for isolation of cfDNA was limited and due to low concentrations only minute amounts cfDNA could be analyzed. This mixture was loaded on digital PCR chips containing 20,000 wells, and cycled under standard conditions for 40 cycles. The QuantStudio™ 3D analysisSuite™ was used to analyze the end-point fluorescence data and to determine the proportion of templates with and without a mutation. Each sample was analyzed in duplicate experiments, and only those with mutant copies detected in both experiments were called mutant.

Prediction pathogenicity of single nucleotide substitutions

Exonic SNVs were evaluated with different *in silico* prediction tools to calculate the pathogenic effect of the SNS on the function of the protein. Part of the tools applied were embedded in ANNOVAR and included SIFT, PolyPhen2, MutationTaster, FATHMM, GERP++, SiPhy and PhyloP [9, 10]. In addition, software packages KD4v and PROVEAN were also applied. Literature searches were performed to obtain, if available, extra functional data.

CALCULATIONS

Variant frequencies were established by NGS and digital PCR. These variant frequencies were used to calculate the number of mutant copies per 1 ng DNA for primary tumor specimens and the number of mutant copies per 1 ml serum for blood specimens. For the calculations, we used the mass of the human haploid genome (approximately 3.3 pg) as indicated previously [11], (http://www3.appliedbiosystems.com/cms/groups/mcb_marketing/documents/generaldocuments/cms_042486.pdf), for 1 DNA molecule to estimate the number of mutant copies by the formulas:

For primary tumor specimens:

*Mutant copies per 1 ng DNA = [Total DNA quantity in ng * 1000/3.3] * [variant frequency/100].*

And for blood specimens:

*Mutant copies per 1ml serum = [Total DNA yield in ng (per 1 ml serum) * 1000/3.3] * [variant frequency/100].*

DETAILS OF RESULTS

The analysis of 10 MBC patients revealed 477 exonic variants in diseased DNA which were not present in sequenced normal DNA or in the 1000 Genome database. At disease progression, 186 variants (Supplementary Table A3) were identified including 47 variants also seen in an additional specimen for a particular patient. The 47 variants included 18 (non)-frameshifts, 10 synonymous SNVs, 3 stop-gain SNVs, and 16 non-synonymous SNVs. All (non)-frameshifts were discarded as artefacts because they occurred in sequence repeats which are prone to sequencing errors. In addition, 11 SNVs were excluded because the sequenced reads failed our quality selection criteria or detected very commonly, i.e. seen in more than 20 specimens at similar (low) frequencies in both tumor and blood specimens, suggestive for a technical artefact. This resulted finally in 18 DNA changes at disease progression, of which 15 variants originated from the primary tumor and 3 variants only seen in blood specimens. The tumor-specific variants contained 6 variants present in all specimens of a particular patient, making these variants less likely to be associated with disease progression. Nine tumor-specific variants were observed at progression and in the primary tumor but not in all blood specimens of earlier time-points. These 9 variants and 3 blood-specific variants were selected as markers that associate with disease progression and evaluated for their pathogenicity scores (Supplementary Table A4).

No trend (P-values all above 0.31) was observed between patients with (patients 1 & 6–10) and without mutational changes (patients 2–5) in the blood in relation to PFS and to time intervals between the different biopsies (Supplementary Table A8).The missense mutations were verified by deeper re-sequencing and *PIK3CA* mutations were additionally verified by digital PCR (Supplementary Table A5).

REFERENCES

1. Berns EM, Klijn JG, van Putten WL et al. c-myc amplification is a better prognostic factor than HER2/neu amplification in primary breast cancer. Cancer Res 1992; 52:1107–1113.
2. van Lier MG, Wagner A, van Leerdam ME et al. A review on the molecular diagnostics of Lynch syndrome: a central role for the pathology laboratory. J Cell Mol Med 2010; 14:181–197.
3. Hiltemann S, Mei H, de Hollander M et al. CGtag: complete genomics toolkit and annotation in a cloud-based Galaxy. Gigascience 2014; 3:1.
4. Wang K, Li M, Hakonarson H. ANNOVAR: functional annotation of genetic variants from high-throughput sequencing data. Nucleic Acids Res. 2010; 38:e164.
5. Goecks J, Nekrutenko A, Taylor J, Galaxy T. Galaxy: a comprehensive approach for supporting accessible, reproducible, and transparent computational research in the life sciences. Genome Biol. 2010; 11:R86.
6. Blankenberg D, Von Kuster G, Coraor N et al. Galaxy: a web-based genome analysis tool for experimentalists. Curr Protoc Mol Biol. 2010; 19:10 11–21.
7. Giardine B, Riemer C, Hardison RC et al. Galaxy: a platform for interactive large-scale genome analysis. Genome Res. 2005; 15:1451–1455.
8. Robinson JT, Thorvaldsdottir H, Winckler W et al. Integrative genomics viewer. Nat Biotechnol. 2011; 29:24–26.
9. Liu X, Jian X, Boerwinkle E. dbNSFP: a lightweight database of human nonsynonymous SNPs and their functional predictions. Hum Mutat. 2011; 32:894–899.
10. Liu X, Jian X, Boerwinkle E. dbNSFP v2.0: a database of human non-synonymous SNVs and their functional predictions and annotations. Hum Mutat. 2013; 34:E2393–2402.
11. Dolezel J, Bartos J, Voglmayr H, Greilhuber J. Nuclear DNA content and genome size of trout and human. Cytometry A 2003; 51:127–128; author reply 129.

Supplementary Table S1: 186 variants identified at disease progression. The table shows the 186 variants that were discovered in at least 1 serum sample taken at disease progression. Details are presented in the table. The 47 variants at disease progression and called in one additional sample for a particular patient are also indicated, as well as the final 18 DNA changes that fulfilled all quality selection criteria.

Supplementary Table S2: Pathogenicity scores of the 12 progression markers. The table shows details for the 12 progression markers and their pathogenicity scores as predicted by the different software tools in ANNOVAR. The highest number of pathogenic SNVs were predicted by the MutationTaster.

Supplementary Table S3: Re-sequencing and digital PCR analyses of missense SNVs. Ion-PGM re-sequencing and dPCR results are presented. All specimens were re-sequenced after independent library preparation for 6 amplicons harboring non-synonymous SNVs. Mutant and total number of reads are shown for all specimens and mutation frequencies for only those higher than 1%. The *PIK3CA* genotype was verified by dPCR for p.H1047L in all specimens of patients 7 and 10 and for p.H1047R in all primary tumors and in blood specimens of patients 1 and 2. Mutant and total number copies are presented; frequencies are only shown when mutant copies were detected in two independent experiments.

Supplementary Table S4: Patient and tumor characteristics

**Clinicopathological factors:**	**Patient 1**	**Patient 2**	**Patient 3**	**Patient 4**	**Patient 5**	**Patient 6**	**Patient 7**	**Patient 8**	**Patient 9**	**Patient 10**
pat_id	p7	p21	p8	p10	p16	p18	p3	p12	p20	p24
At diagnosis of early disease:										
Age	64	50	43	43	48	58	62	51	52	61
Menopausal status	postmenopausal	premenopausal	premenopausal	premenopausal	premenopausal	postmenopausal	postmenopausal	premenopausal	premenopausal	postmenopausal
Lymph node status	positive	positive	positive	positive	positive	positive	positive	negative	negative	positive
Tumor size	≤ 20 mm	≤ 20 mm	> 20 mm	≤ 20 mm	> 20 mm	> 20 mm	> 20 mm	≤ 20 mm	> 20 mm	≤ 20 mm
Histology	IDC	IDC	IDC	Unknown	DCIS+IDC	DCIS+IDC	IDC	IDC	IDC	IDC
Grade	unknown	poor	poor	poor	good/moderate	poor	poor	poor	poor	good/moderate
Progesteron receptor status	positive	positive	negative	positive	positive	negative	positive	positive	positive	positive
Her2 status	unknown	unknown	negative	negative	positive	positive	unknown	unknown	unknown	negative
Treatment early disease:										
Surgery	mastectomy	breast conserving	breast conserving	breast conserving	mastectomy	mastectomy	breast conserving	breast conserving	mastectomy	mastectomy
Radiotherapy	no	yes	yes	yes	no	yes	yes	yes	no	yes
Adjuvant chemotherapy	anthra	n-anthra	n-anthra	n-anthra	anthra	n-anthra	no	no	no	no
Clinical outcome:										
Overall survival (in months)	134	113	187	177	177	87	108	127	71	123
Metastasis-free survival (in months)	96	55	124	107	52	54	87	105	29	69
At diagnosis of advanced disease:										
Age	72	54	53	52	52	63	69	60	54	66
Menopausal status	postmenopausal	postmenopausal	postmenopausal	premenopausal	postmenopausal	postmenopausal	postmenopausal	postmenopausal	premenopausal	postmenopausal
First-line tamoxifen treatment outcome:										
Progression-free survival (in months)	6	13	24	36	13	22	15	15	6	25
Response	SD ≤ 6 m	SD > 6 m	SD >6 m	SD > 6 m	PR	SD > 6 m	SD > 6 m	SD > 6 m	SD ≤ 6 m	SD > 6 m
Determination of disease progression	Bone Scan	CT-Abdomen: livermeta	Bone Scan & X-ray skeleton	X-Ray and MRI pelvis	Increase SC-lymph nodes	Bone Scan	X-ray thorax and pelvis; Bone Scan	CT-Brain/Abdomen: Brain &liver meta	Bone Scan	MRI-twk
Yield and concentration cfDNA isolated from 400 µl serum taken at:										
before/at start tamoxifen	6.7 ng (228 ng/ml)	8.6 ng (87 ng/ml)	5.7 ng (70 ng/ml)	6.2 ng (65 ng/ml)	19.2 ng (200 ng/ml)	9.2 ng (96 ng/ml)	5.9 ng (64 ng/ml)	7.0 ng (74 ng/ml)	13.6 ng (139 ng/ml)	2.7 ng (27 ng/ml)
during treatment	6.8 ng (70 ng/ml)	10.5 ng (107 ng/ml)	5.7 ng (92 ng/ml)	6.0 ng (63 ng/ml)	4.4 ng (61 ng/ml)	11.9 ng (127 ng/ml)	2.9 ng (32 ng/ml)	9.7 ng (102 ng/ml)	8.4 ng (86 ng/ml)	4.2 ng (43 ng/ml)
at disease progression	6.5 ng (66 ng/ml)	12.2 ng (124 ng/ml)	5.8 ng (80 ng/ml)	6.3 ng (67 ng/ml)	9.0 ng (113 ng/ml)	9.6 ng (99 ng/ml)	7.0 ng (76 ng/ml)	45.5 ng (484 ng/ml)	4.3 ng (44 ng/ml)	4.1 ng (57 ng/ml)

The table presents the clinicopathological factors of the 10 metastatic breast cancer patients who received first-line tamoxifen which were selected for this study. Additionally, the yields and concentrations of isolated cfDNA at the different time-points are presented. Used abbreviations: IDC= invasive ductal carcinoma; DCIS= ductal carcinoma in situ; anthra= anthracycline based chemotherapy; SD= stable disease.

Supplementary Table S5: Ion–PGM input, genomic equivalents, and limit of detection

Patient ID	Sample	SampleID	DNA concentration (pg/ul)	amount DNA in library preparation (in pg)	amount DNA library pool sequenced (in pM)	Genomic equivalents	Limit of Detection
1	Ss	JH18	228	395	458	65	2%
	St	KE38	69.8	395	164	65	2%
	Sp	KV34	65.8	395	100	65	2%
2	Ss	LW69	87.4	524	115	86	1%
	St	OC98	106.8	524	157	86	1%
	Sp	OM84	124.4	524	133	86	1%
3	Ss	EV61	70	420	367	69	1%
	St	JS80	92	420	352	69	1%
	Sp	KM100	80	420	683	69	1%
4	Ss	FW8	64.8	379	185	62	2%
	St	NJ32	63.2	379	405	62	2%
	Sp	OC8	67.2	379	640	62	2%
5	Ss	EZE9	200	367	813	60	2%
	St	JJ43	61.2	367	587	60	2%
	Sp	KB81	113	367	473	60	2%
6	Ss	HC35	95.6	574	623	94	1%
	St	LM35	126.8	574	746	94	1%
	Sp	MP34	99	574	679	94	1%
7	Ss	GB45	63.6	250	190	41	2%
	St	JG94	31.8	191	128	31	3%
	Sp	KA38	75.8	250	313	41	2%
8	Ss	LK98	74.2	445	699	73	1%
	St	OF40	102.2	445	809	73	1%
	Sp	OO93	484	445	758	73	1%
9	Ss	DK57	139.4	265	299	43	2%
	St	DW7	86.2	265	377	43	2%
	Sp	EJ44	44.2	265	341	43	2%
10	Ss	BT30	27.6	166	78	27	4%
	St	FG97	43	250	115	41	2%
	Sp	FZ20	57.8	250	183	41	2%

The amounts of cfDNA used as input for the sequencing library preparation (in pg) and sequenced (in pM) by ion-PGM are presented for each blood sample. Based on these amounts the genomic equivalents and limit of detection were calculated. Additionally, the table presents the DNA concentrations (in pg/ul) for the blood biopsies (Ss, St, Sp).

Supplementary Table S6: Digital PCR input, genomic equivalents, and limit of detection. The DNA input amounts (in pg) evaluated by digital PCR are presented for primary tumor (pT) and blood biopsies (S1-S4, Ss, St, Sp) based on Qubit measurements and digital PCR calculations. These input amounts were used to establish the number of genomic equivalents that subsequently defines for each sample the limit of detection (LoD). Additionally, the table presents the number mutant (FAM-labeled) and wildtype (VIC-labeled) copies per microliter, and mutant allele frequency with its 95% confidence interval originally measured by digital PCR. The MAF in red indicate those that are below both LoDs. Digital PCR analyses were performed in duplicate for all specimens, except pT of patient 1.

Supplementary Table S7: Ion-PGM custom gene panel. The table presents information for the 45 genes selected in this study for targeted ion-PGM sequencing. It shows for each gene, amongst other details, the number of amplicons and covered bases sequenced, the in COSMIC release 67 reported mutation frequencies observed for breast, colon, prostate, and ovarian cancer. The table indicates also core pathways and available targeted drugs reported in www.drugbank.ca .

Supplementary Table S8: Biopsy time intervals

Patient	Specimen #	Biopsy date	Follow-up	Biopsy time intervals (in months)		
			Response to tamoxifen @	**Tissue (pT)-liquid (Ss) biopsy**	**First (Ss)-Second (St) liquid biopsy**	**Second (St)-Third (Sp) liquid biopsy**
1	pT	July, 1987	9 years			
	Ss	June, 1995	6 months SD	95		
	St	October, 1995			4	
	Sp	January, 1996				3
2	pT	August, 1990	7 years			
	Ss	June, 1996	13 months SD	70		
	St	June, 1997			12	
	Sp	August, 1997				3
3	pT	July, 1983	12 years			
	Ss	December, 1993	24 months SD	125		
	St	August, 1995			20	
	Sp	December, 1995				5
4	pT	June, 1985	12 years			
	Ss	May, 1994	36 months SD	107		
	St	February, 1997			33	
	Sp	June, 1997				5
5	pT	May, 1990	5 years			
	Ss	October, 1994	13 months PR	54		
	St	June, 1995			8	
	Sp	October, 1995				5
6	pT	May, 1990	6 years			
	Ss	December, 1994	22 months SD	56		
	St	April, 1996			16	
	Sp	October, 1996				7

7	pT	March, 1987	8 years			
	Ss	June, 1994	15 months SD	87		
	St	June, 1995			12	
	Sp	September, 1995				3
8	pT	July, 1987	10 years			
	Ss	April, 1996	15 months SD	104		
	St	June, 1997			13	
	Sp	August, 1997				3
9	pT	August, 1990	3 years			
	Ss	March, 1993	6 months SD	31		
	St	June, 1993			3	
	Sp	September, 1993				3
10	pT	October, 1986	8 years			
	Ss	June, 1992	25 months SD	32		
	St	January, 1994			19	
	Sp	June, 1994				6

Abbreviation for biopsy specimen: Primary tumor tissue (pT), serum at start of therapy (Ss), serum during treatment (St), and serum at disease progression (Sp).
@ Response to first-line tamoxifen assessed by RECIST criteria and defined as stable disease (SD) or partial response (PR). Information on biopsy date and response to tamoxifen for each patient is presented. Time intervals (in months) were determined for time between tumor tissue (pT) and first liquid (Ss) biopsy, between first (Ss) and second (St) liquid biopsy, and between second (St) and third (Sp) liquid biopsy.

Network 1 – Cell Cycle

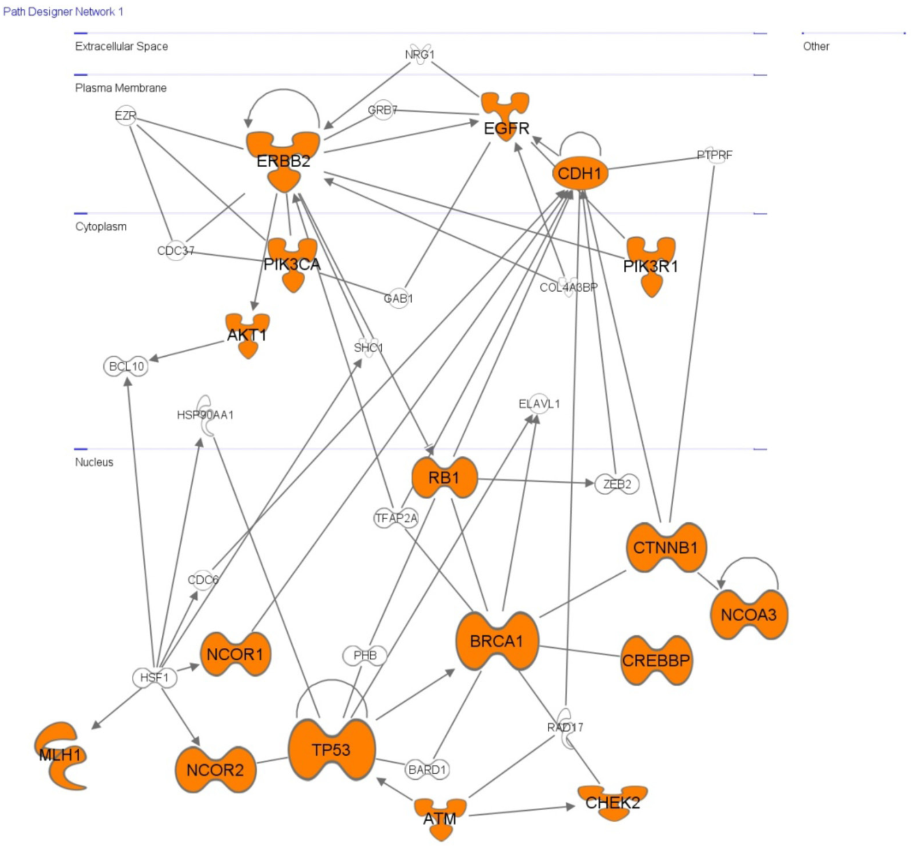

Network 2 – Gene expression

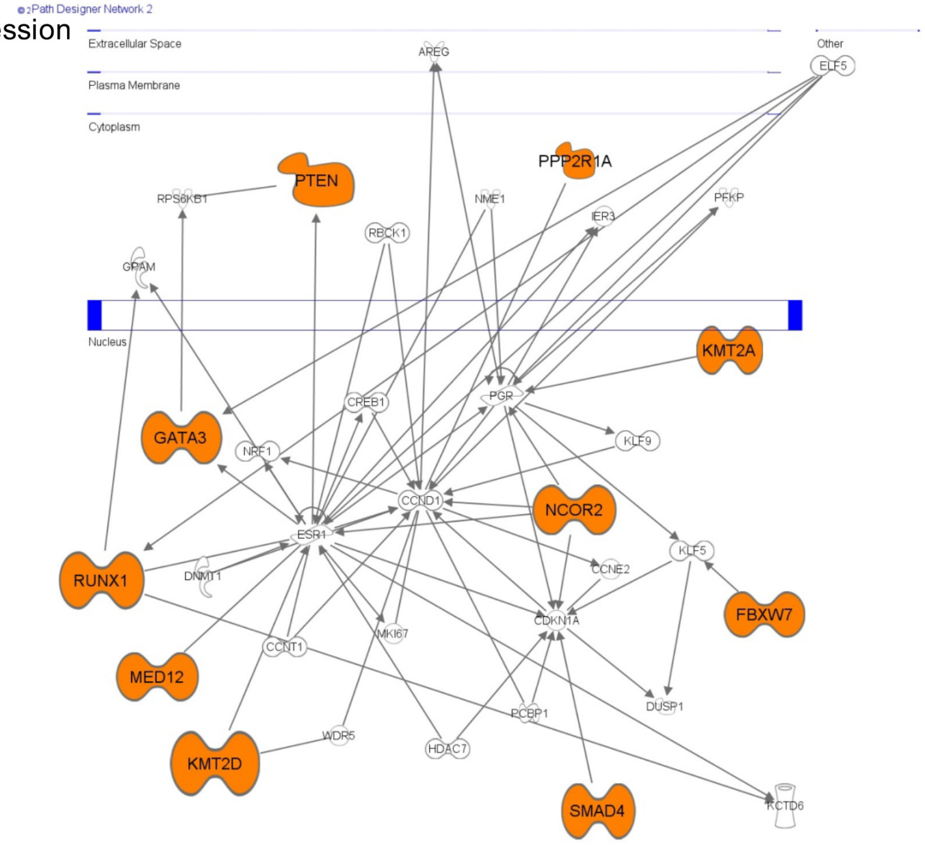

Supplementary Figure S1: Pathway analysis 45 gene panel. Ingenuity Pathway analysis was used to evaluate the 45 genes for their relationships, networks and involved pathways. Two networks were identified, i.e. network 1 included genes involved in cell cycle and network 2 containing genes that play a role in gene expression regulation.
